# Integrated proteome and malonylome analyses reveal the potential meaning of TLN1 and ACTB in end-stage renal disease

**DOI:** 10.1186/s12953-023-00211-y

**Published:** 2023-10-13

**Authors:** Ruqi Tan, Dandan Li, Nan Hu, Jing Qiu, Zhipeng Zeng, Wanxia Cai, Yafang Zhong, Xinzhou Zhang, Pearl Pai, Kang Wang, Donge Tang, Yong Dai

**Affiliations:** 1 Clinical Medical Research Center, The Second Clinical Medical College of Jinan University (Shenzhen People’s Hospital), Shenzhen, Guangdong 518020 P.R. China; 2 Department of Nephrology, The University of Hong Kong-Shenzhen Hospital, Shenzhen, 518053 Guangdong China; 3 Experimental Center, Shenzhen Pingle Orthopedic Hospital (Shenzhen Pingshan Traditional Chinese Medicine Hospital), Shenzhen, Guangdong 518118 China; 4 Key Renal Laboratory of Shenzhen, Department of Nephrology, The Second Clinical Medical College of Jinan University (Shenzhen People’s Hospital), Shenzhen, 518020 Guangdong China; 5 The First Affiliated Hospital, School of Medicine, Anhui University of Science and Technology, Huainan, Anhui 232001 China

**Keywords:** Lysine malonylation (Kmal)_1_, Post-translational modification (PTM)_2_, Chronic kidney disease (CKD)_3_, End-stage renal disease (ESRD)_4_, Platelet activation_5_

## Abstract

**Background:**

End-stage renal disease (ESRD) is a condition that is characterized by the loss of kidney function. ESRD patients suffer from various endothelial dysfunctions, inflammation, and immune system defects. Lysine malonylation (Kmal) is a recently discovered post-translational modification (PTM). Although Kmal has the ability to regulate a wide range of biological processes in various organisms, its specific role in ESRD is limited.

**Methods:**

In this study, the affinity enrichment and liquid chromatography-tandem mass spectrometry (LC-MS/MS) techniques have been used to create the first global proteome and malonyl proteome (malonylome) profiles of peripheral blood mononuclear cells (PBMCs) from twenty patients with ESRD and eighty-one controls.

**Results:**

On analysis, 793 differentially expressed proteins (DEPs) and 12 differentially malonylated proteins (DMPs) with 16 Kmal sites were identified. The Rap1 signaling pathway and platelet activation pathway were found to be important in the development of chronic kidney disease (CKD), as were DMPs TLN1 and ACTB, as well as one malonylated site. One conserved Kmal motif was also discovered.

**Conclusions:**

These findings provided the first report on the Kmal profile in ESRD, which could be useful in understanding the potential role of lysine malonylation modification in the development of ESRD.

**Supplementary Information:**

The online version contains supplementary material available at 10.1186/s12953-023-00211-y.

## Background

Chronic kidney disease (CKD) has been identified as a prevalent disease in the general population. It has a global estimated prevalence of 13.4% (11.7–15.1%) [1–3], which is defined as a decrease in estimated glomerular filtration rate (eGFR) below 60 mL/min/1.73 m^2^ or the presence of kidney damage for at least three months [4, 5]. Individuals with CKD have an increased risk of hospitalization, anemia, coagulation abnormalities [6], mineral and bone disorders, cardiovascular events [7], and stroke [8]. CKD is further classified into five stages (I–V) based on eGFR. CKD V, the final stage of CKD, refers to patients with an eGFR less than 15 mL/min/1.73 m^2^ regardless of the need for renal replacement therapy (RRT), while ESRD requires RRT [9]. In recent years, ESRD has been seen as a major public health problem due to its increasing frequency of morbidity and mortality, high medical expenses, and also the substantial economic burden it generates [10, 11]. The prevalence and treatment status of ESRD require a further understanding of this disease.

ESRD is characterized by chronic inflammation or a pro-inflammatory milieu along with a concomitantly impaired immune system [12, 13]. Persisted platelet dysfunction is always observed in renal pathology, which might result in an increased predisposition to both bleeding risk and thrombotic complications in patients with ESRD [14, 15]. As expected, there were strong correlations between inflammatory status and impaired coagulation disorders in this group of patients [16]. The accumulation of uremic toxins in patients with ESRD stimulates platelet abnormalities, which are believed to be the cause of the risk of bleeding that most patients face [14]. Meanwhile, blood disorders, comorbidities, inflammation, and endothelial dysfunction are a few additional risk factors that can contribute to thrombotic events in the ESRD group of individuals [15]. Although chronic inflammation and abnormal platelet function have been implicated in the development of ESRD, there are underlying mechanisms that need to be completely clarified.

Post-translational protein modifications (PTMs) represent an essential processing segment in protein formation. They are involved in various biological processes and a variety of human diseases [17, 18]. Aberrant PTMs have been reported to contribute to CKD or chronic renal disease [19, 20]. Lysine 2-hydroxyisobutyrylation had been identified as key regulatory roles in IgA nephropathy [21]. Histone lysine crotonylation had also been investigated in kidney injury [22]. Lysine crotonylation was also found to play an important regulatory role in the pathophysiology of patients on maintenance hemodialysis (MHP) [23]. Meanwhile, histone acetylation has been extensively studied in acute kidney injury, renal fibrosis [20], diabetic nephropathy [24], and CKD [25]. These studies suggest that PTMs are involved in the pathology of kidney diseases.

Lysine malonylation (Kmal), a novel type of PTM, was first discovered in 2011. At the same time, a regulatory enzyme [26] that catalyzes lysine malonylation, Sirt5 [26], was reported. It was then revealed that Sirt5, a global regulator of lysine malonylation, regulates the energy cycle through glycolysis [27]. Du and co-workers detected more malonylated lysine sites and proteins in type 2 diabetes mice than in wild-type (WT) mice. Bioinformatic analysis of malonylated proteins and validation of fructose bisphosphate aldolase B (ALDOB) function reveals that glucose and lipid metabolism disorders in type 2 diabetes may be related to dysfunction of key enzymes resulting from aberrant PTM [28]. Protein malonylation was first identified as an inflammatory signal that regulated the binding between GAPDH and its target mRNA [29]. Inspired by the functions of Kmal in diabetes and inflammation, which are closely related to the onset and progression of CKD, this study is interested in the potential role of Kmal in kidney disease.

This study used LC-MS/MS in conjunction with a sensitive immune affinity purification technique to characterize proteomic and malonylomic proteins in patients with ESRD. In order to analyze the data obtained and investigate the pathogenic mechanisms of ESRD, elaborate bioinformatic strategies, such as Gene Ontology (GO) analysis, Kyoto Encyclopedia of Genes and Genomes (KEGG) signaling pathway analysis and motif analysis, were utilized. According to functional analyses, malonylated proteins were found to be prevalent in the immune system process, integrin-mediated signaling pathway, Rap1 signaling pathway, and platelet activation. This is the first study to characterize integrated proteome and malonylome analysis in ESRD. The findings discovered in this research could pave the way for further studies on the pathogenic activities of malonylated proteins in ESRD.

## Materials and methods

### Patients and PBMCs isolation

Peripheral blood samples were drawn from 20 patients with ESRD and 81 control subjects with normal kidney function between May 2020 and October 2020. According to the 2012 Kidney Disease Improving Global Outcomes Organization (KDIGO) Clinical Practice Guideline for the Evaluation and Management of Chronic Kidney Disease [30], all patients were confirmed with the diagnosis of ESRD (eGFR less than 15 mL/min/1.73 m^2^) and were also on RRT (hemodialysis or peritoneal dialysis) at Shenzhen People’s Hospital. The primary causes of renal failure were diabetic nephropathy (*n =* 4), immunoglobulin A (IgA) nephropathy (*n =* 1), and glomerulonephritis (*n =* 15). Eighty-one control subjects without kidney disease and without administering drugs were included in the study. All participants with active infections, a history of cancer, inflammatory, autoimmune, hematologic, or allergic diseases were excluded from the study. The Shenzhen People's Hospital Medical Ethics Committee approved all experimental procedures, which were carried out in accordance with Chinese laws and regulations. Before the study, all participants signed a written informed consent form. The processes involved in this study were carried out on the basis of the Declaration of Helsinki.

EDTA anticoagulated blood (about 8 ml) was the sample used for the isolation of peripheral blood mononuclear cells (PBMC) through Ficoll-Hypaque density gradient centrifugation. Following centrifugation and isolation of the buffy coat, the PBMCs were isolated from blood by layering the diluted blood with phosphate buffered saline (PBS) (1:1 in PBS) on top of an equal volume of Ficoll (GE Healthcare). After PBMC isolation, these samples were stored at -80 ° C until further analysis was performed.

### Protein extraction

Once the stored PBMC samples were removed from-80°C, four volumes of lysis buffer (containing 8 mol / L of urea, 1% protease inhibitors, 3 µM TSA, and 50 mM NAM) were added to the unwashed sample, followed by ultrasonic sonication (PTM Bio, Hangzhou, China) and centrifugation (12000 ×g at 4 ° C for 10 min) to remove cell debris. Next, the supernatants were then transferred to a new microcentrifuge tube and collected for protein concentration assessment, which was determined using a BCA kit (Beyotime Biotechnology) according to the manufacturer's instructions.

### Trypsin digestion

An equal amount of each sample was taken for enzymatic hydrolysis, and the volume was adjusted to the same with the lysis solution. After collection, the samples were subjected to precipitation of trichloroacetic acid (TCA, Sigma-Aldrich) precipitation at a final concentration of 20% and incubated at 4 °C for 2 h, followed by centrifugation (4500 ×g, 5 min). In the next steps, the supernatant was discarded, and the remaining precipitate was washed with cold acetone about two to three times. Triethylammonium bicarbonate buffer (final concentration: 200 mM TEAB, Sigma) was then added to completely dissolve the sample, and trypsin (Promega) was added at a mass ratio of 1:50 (enzyme: protein) and kept at 37°C overnight. The proteins were then reduced by adding dithiothreitol (DTT) (final concentration: 5 mM) for 30 min at 56°C, followed by the addition of iodoacetamide (IAA) to a final concentration of 11 mM and further incubated for 15 min in the dark at room temperature.

### Affinity enrichment of lysine malonylated peptides

The peptide was dissolved in the IP buffer solution (100 mM NaCl, 1 mM EDTA, 50 mM Tris-HCl, 0.5% NP-40, pH 8.0), and the supernatant was transferred to the malonylated resin that was washed in advance (antibody resin item number PTM-904, Hangzhou Jingjie Biotechnology Co., Ltd., PTM Bio). The samples were placed in a rotating 4 °C shaker, gently shaken and incubated overnight. After the incubation, the resin was washed four times with IP buffer solution and twice with deionized water. Finally, 0.1% trifluoroacetic acid was used to elute the resin-bound peptides; the process was repeated three times. The eluent was discarded and samples were desalinated following the instructions given with C18 ZipTips (Merck Millipore). Finally, the peptide samples were freeze dried and used for LC/MS analysis.

### LC-MS/MS analysis

The peptides were then dissolved in mobile phase A of liquid chromatography, and the mixture were separated with the NanoElute ultrahigh-performance liquid system (Bruker, Karlsruhe, Germany), packed with 1.9 μm/120 Å ReproSilPurC18 resins(Dr. Maisch GmbH, Ammerbuch, Germany) [31]. Mobile phase A was 0.1% formic acid with 2% acetonitrile, mobile phase B was 0.1% formic acid with 100% acetonitrile. In proteomic analysis, the gradient of mobile phase B was raised from 6% to 24% in 70 min, 24% to 32% within 14 min, 32 to 80% in 3 min, then maintained at 80% in 3min. In the malonylome analysis, the gradient of mobile phase B was raised from 6% to 24% in 42 min, 24% to 32% within 14 min, 32% to 80% in 2 min, then maintained at 80% in 2min. After the separation and ionization of peptides using an ultrahigh-performance liquid system and a capillary ion source, we utilized timsTOF Pro mass spectrometry (Bruker, Karlsruhe, Germany)to analyze the peptides. Next, the ion source voltage was set to 1.6/1.7 kV. For the MS scans, the m/z scan range was adjusted between 400 and 1500. The data acquisition mode was based on the parallel accumulation serial fragmentation (PASEF) mode. After collecting a first-level mass spectrum, the second-level spectrum with parent ion charges in the range of 0-5 were collected in PASEF mode 10 times, and the dynamic exclusion time of the tandem mass spectrometry scan was set at 30 s to avoid repeated sequencing of identical precursor ions.

### Database search

The acquired MS raw files were processed with the MaxQuant search engine (v.1.6.15.0). The MS/MS spectra were then searched against the Homo sapiens protein database (Homo_sapiens_9606_SP_20201214. with 20395 sequences) in SwissProt that reversed protein sequences were used as decoys. We calculated the label-free quantification (LFQ) for protein quantification in this study. The digestion method Trypsin/P was set, and four missed cleavage sites were allowed. The mass tolerance for precursor and fragment ions was set at 20 ppm in the first search and in the main search, and the error tolerance of the mass of the secondary fragment ion was to 20 ppm. The minimum peptide length was set to seven amino acids, with a maximum of five modifications per peptide. Methionine oxidation, protein N-terminal acetylation, and lysine malonylation were established as the variable modifications, while the cysteine alkylation was specified as a fixed modification. The false discovery rate (FDR) for peptides, protein identifications, and modification sites was set to 1%. Most peptides were distributed in the range of 7-20 amino acids. The proteins were identified by a minimum of two peptides and at least one unique peptide. These common parameters were used to analyze searches in the proteome and malonylome databases.

### Bioinformatics analysis

For the Gene ontology (GO) annotation analysis, identified proteins were annotated utilizing eggNOG-mapper software (v2.0) ( https://github.com/eggnogdb/eggnog-mapper) [32]. At the same time, protein domains were annotated with the Pfam database (v33.1) using the PfamScan software. PSORTb software (v3.0) was also performed to predict the subcellular location of the submitted proteins. For the functional enrichment analysis, a two-tailed Fisher’s exact test was used to test differentially expressed proteins in the background of identified proteins. The gene ontology (GO) annotation category with a corrected *P*-value < 0.05 is considered significant. Moreover, Encyclopedia of Genes and Genomes (KEGG) mapper (v2.5) (https://www.kegg.jp/kegg/mapper/) [33] was used to identify enriched pathways by a two-tailed Fisher’s exact test to test the enrichment of the differentially expressed protein against all identified proteins. The pathway with a corrected *P*-value < 0.05 was considered significant. This study also used MoMo analysis tool (v5.0.2) (https://meme-suite.org/meme/tools/momo) [34], a motif-x algorithm, to analyze the model of the sequences of malonylated peptides, which contain amino acids at a specific position of the malonyl 21-mer in all protein sequences (10 amino acids upstream and downstream of the malonylation site).

## Results

### Clinical characteristics

Peripheral blood was drawn from 20 dialysis patients and 81 controls with normal kidney function. There were 20 patients with ESRD, of whom 10 were men and 10 women, with an average age of 50.2 years. At the same time, all patients with ESRD with an eGFR less than 15 mL/min / 1.73 m are admitted to RRT (hemodialysis or peritoneal dialysis). The basic clinical information of ESRD patients is shown in Table 1.

**Table 1:** The clinical characteristics of end-stage renal disease (ESRD) patients

	Gender	Age	SCR (umol/l)	BUN (mmol/l)	eGFR (ml/min*1.73 m^2^)
1	male	33	1569.2	29.68	3.04
2	female	50	937	18.36	3.79
3	male	35	1209.8	20.64	3.37
4	female	63	1189	35.5	2.60
5	female	53	938	19.96	3.71
6	male	56	1384	30.1	3.01
7	female	55	715	28.43	5.08
8	male	41	1305	27.96	3.59
9	male	58	1396.4	21.62	2.94
10	male	39	1210	22.52	3.99
11	male	47	1623.5	26.64	2.65
12	male	40	980	17.64	5.11
13	female	47	644	12.58	6.10
14	male	74	1047	26.3	3.72
15	male	38	926	16.2	5.56
16	female	57	1104	46.56	2.96
17	female	63	818	32.55	4.08
18	female	52	837	18.5	4.29
19	female	61	886	33.1	3.76
20	female	42	1060	25.75	3.46

### Qualitative and quantitative proteomic profiling and proteomic analysis

**Fig. 1:**
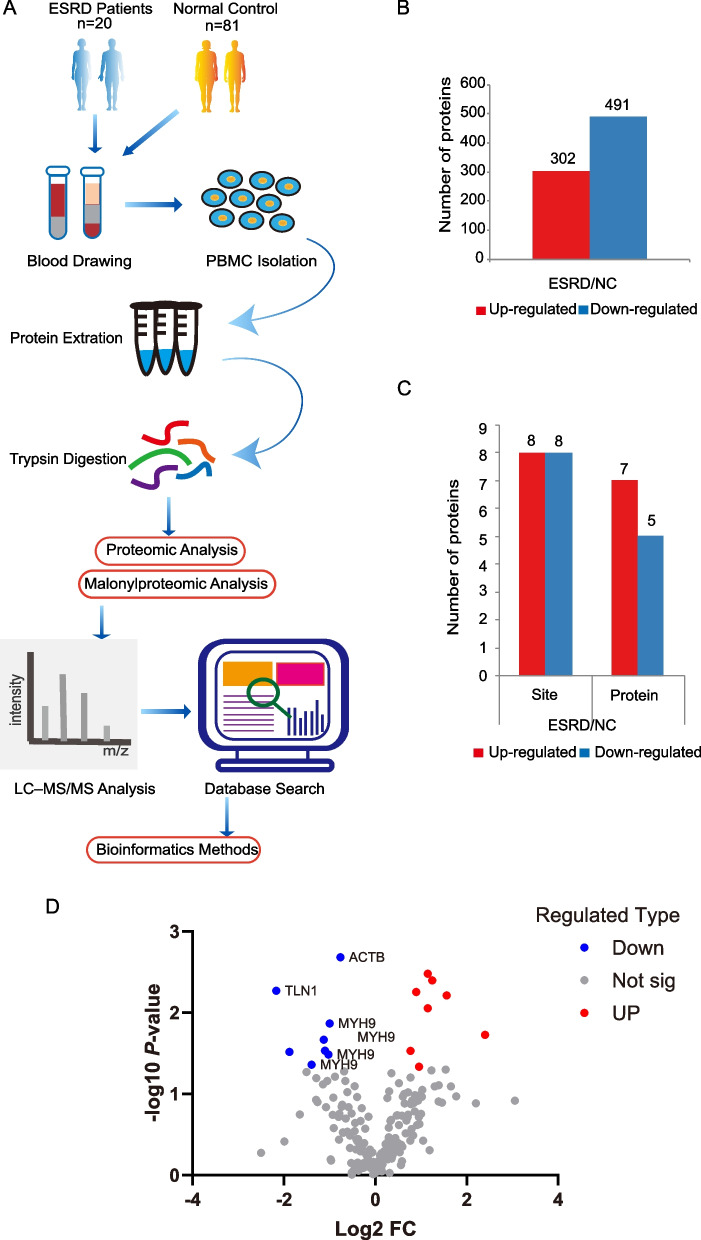
The schematic flow to study the proteome and malonylproteome of peripheral blood mononuclear cells (PBMCs) in End-stage renal disease (ESRD) patients and normal controls (NCs). **A** Overview of experimental procedures. **B** Histogram of quantity distribution of differentially expressed proteins (DEPs). Of these 793 DEPs, 302 DEPs were found to be up-regulated and 491 DEPs were down-regulated when compared with the controls. **C** Histogram of quantity distribution of differentially malonylated proteins (DMPs). 16 malonylation sites were identified in 12 DMPs, among which 8 sites on 7 proteins were hyper-malonylated, while 8 sites on 5 proteins were hypo-malonylated. (Fold change >1.5 or <1/1.5 and *P*-value <0.05). **D** In the volcano plots displaying DMPs, TLN1, ACTB and MYH9 are labeled

The entire experimental workflow is shown in Fig. 1A. This study identified 34848 unique peptides from a total of 36504 peptides based on a shotgun proteomics technique. Of these 4354 identified proteins, 3383 proteins were quantifiable in ESRD or controls with normal kidney function (Supplementary Figure 1 and Supplementary Table 1). The distribution of peptide number defining each protein was illustrated in Supplementary Figure 2. Most proteins in fact correspond to more than two peptides. Supplementary Figures 3–5 show the distribution of the protein's sequence coverage, the peptide sequence length, and the protein mass of the identified protein, respectively. The coverage of most proteins is below 30%. And the protein coverage is positively correlated with abundance in the sample. Regarding protein mass distribution, the molecular weight of the identified proteins was uniformly distributed in different stages. All these met the quality control requirements and suggested high confidence. Quantified proteins with a fold change (FC) cutoff >1.5 or < 1/1.5 (0.667) and a *P*-value < 0.05 were considered significant (FC represents the fold change in the ESRD group versus the HC group). As a result, in our study,793 differentially expressed proteins (DEPs) were revealed, of which 302 proteins were found to be up-regulated (FC > 1.5) and 491 proteins were identified as down-regulated (FC < 1/1.5) in ESRD patients (Fig. 1B and Supplementary Table 2).

After lysine malonylation, 130755 spectra were generated and 22122 were matched to known spectra. Among these identified spectra, 6421 malonylated peptides were identified. Finally, this investigation identified 807 malonylation sites assigned to 310 identified malonylated proteins (Supplementary Figure 6). A total number of 406 quantifiable malonylation sites were recognized in 174 quantifiable malonylated proteins. Supplementary Figure 7 shows the peptide length distribution of the identified malonylated proteins. According to the screening criteria (fold change >1.5 or <1/1.5 and *P*-value <0.05), 16 malonylation sites were identified in 12 DMPs. Of the 12 DMPs, 8 sites in 7 proteins were hyper-malonylated (fold change >1.5), while 8 sites in 5 proteins were hypo-malonylated (fold change <1/1.5) (Fig. 1C and Supplementary Table 3). Notably, MYH9 has four Kmal sites (Fig. 1D). According to genome-wide association studies (GWAS), polymorphisms in MYH9 have been reported to be strongly associated with ESRD, including focal segmental glomerulosclerosis (FSGS), global glomerulosclerosis, diabetic nephropathy, and collapsing glomerulopathy [35–37].

### Functional classification of subcellular localization and GO analysis of DEPs and DMPs

**Fig. 2:**
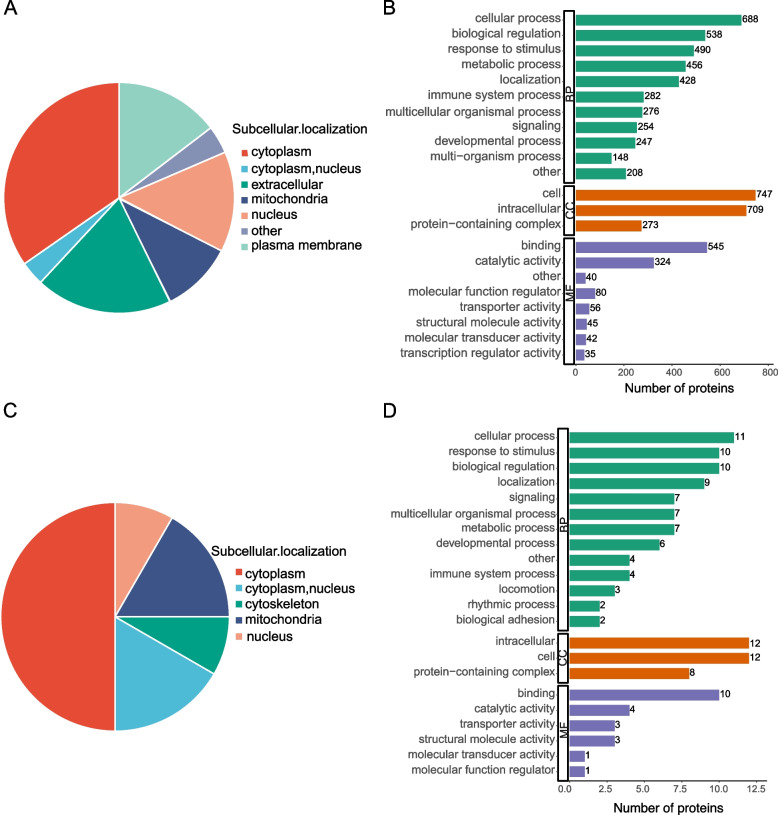
Functional classification of subcellular localization and Gene Ontology (GO) secondary annotation analysis of the differentially expressed proteins (DEPs) and differentially malonylated proteins (DMPs). **A** Subcellular localization of the DEPs. **B** The GO annotation of all DEPs. **C** Subcellular localization of the DMPs. **D** The GO annotation of all DMPs. BP, Biological Process; CC, Cellular Component; MF, Molecular Function

The function and subcellular locations of DEPs and DMPs between the ESRD group and the healthy group were characterized using the GO functional classification (Fig. 2). According to the findings, most DEPs were located in the cytoplasm (*n =* 275, 34.6%), the extracellular space (*n =* 152, 19.2%), and the plasma membrane (*n =* 116, 14.6%) (Fig. 2A). The GO secondary annotation of the DEPs is shown in Fig. 2C. The main categories of biological processes (BP) include biological regulation, cellular processes, immune system processes, response to stimulus, localization, and metabolic processes. The most notable categories of cell components (CC) were intracellular, cell-containing, and protein-containing complexes. The categories of the most essential molecular function (MF) consist of catalytic activity, binding and transporter activity, molecular structure activity, and molecular function regulator categories.

When considering the subcellular location of the DMPs, this study found that most of the DMPs were originally from the cytoplasm (*n =* 6, 50%), cytoplasm and nucleus (*n =* 2, 16.7%), nucleus (*n =* 1, 8.3%), mitochondria (*n =* 2, 16.7%), and cytoskeleton (*n =* 1, 8.3%) (Fig. 2B). The GO annotations of the DMPs revealed that the biological regulation of the BP ontology, the response to stimulus, and the categories of cellular processes, as well as intracellular, cell, and protein-containing complexes in the CC ontology, and catalytic activity and binding in the MF ontology, were the most prevalent (Fig. 2D).

### GO and KEGG functional enrichment analyses of DEPs

**Fig. 3:**
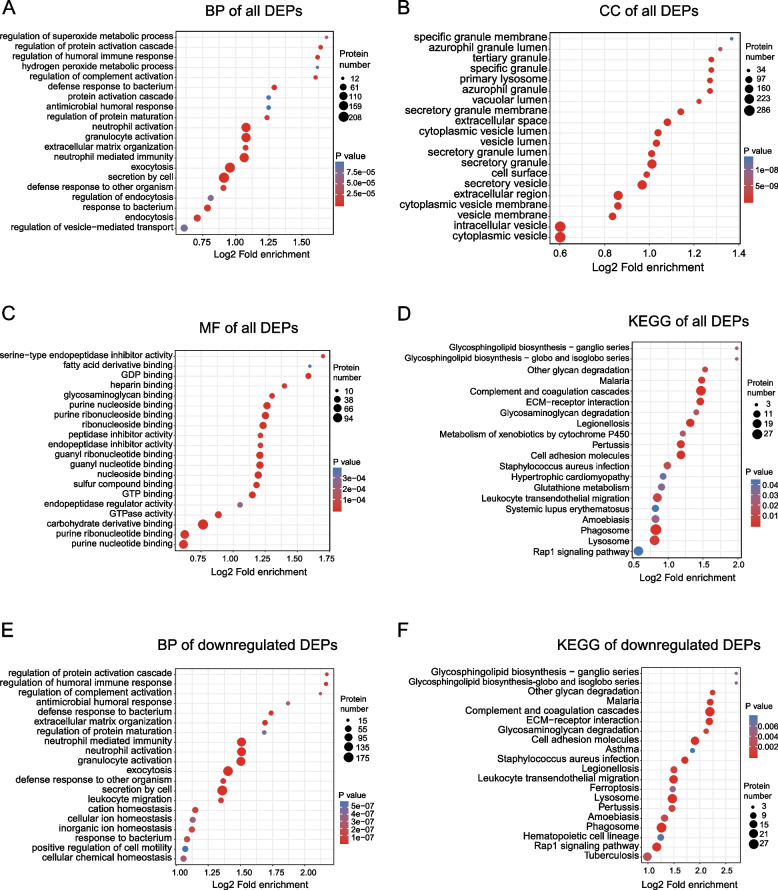
Gene Ontology (GO) and Kyoto Encyclopedia of Genes and Genomes (KEGG) functional enrichment analysis of all differentially expressed proteins (DEPs) and down-regulated DEPs in end-stage renal disease (ESRD) patients compared with normal controls (NCs). **A** Biological process (BP) analysis of all DEPs. **B** Cellular component (CC) analysis of all DEPs. **C** Molecular function (MF) analysis of all DEPs. **D** KEGG functional enrichment analysis of all DEPs. **E** BP analysis of down-regulated DEPs. **F** KEGG functional enrichment analysis of down-regulated DEPs. The size of the circles denotes the number of DEPs enriched in the pathway, while circle color indicates the *P* -value significance (larger circle and red color indicates stronger significance)

To better understand the biological role of these DEPs, this research used enrichment analyses of GO and KEGG (Fig. 3 and Supplementary Table 4). For BP (Fig. 3A), DEPs were mainly enriched in exocytosis, granulocyte activation, regulation of complement activation, regulation of humoral immune response, neutrophil-mediated immunity, and defense response to the bacterium. In the CC category (Fig. 3B), most DEPs were associated with secretory granules and extracellular regions. In the MF category, most DEPs were related to carbohydrate derivative binding, purine ribonucleotide binding, and guanyl nucleotide-binding (Fig. 3C). DEPs were considerably enriched in the leukocyte transendothelial migration (TEM), complement and coagulation cascades, ECM-receptor interaction, malaria, and systemic lupus erythematosus. Finally, the KEGG pathway-based enrichment study indicated that DEP could be related to inflammatory processes and immune responses (Fig. 3D).

Next, this research performed a GO enrichment analysis on down-regulated DEPs and found that these DEPs were most enriched in BP terms related to inflammatory and immune processes, such as neutrophil-mediated immunity, granulocyte activation, regulation of humoral immune response, neutrophil activation, leukocyte migration, and regulation of complement activation (Fig. 3E). Similarly, the KEGG enrichment analysis was performed on the same down-regulated DEPs, and it showed that inflammation-related pathways were the most common, including complement and coagulation cascades, leukocyte transendothelial migration, and the Rap1 signaling pathway (Fig. 3F). It should be noted that DEPs downregulated like ITGB2, a member of the integrin β2 family, are closely involved and play important roles in leukocyte adhesion, immunological, and inflammatory responses [38]. They were involved in these three KEGG pathways. This is consistent with the study by Andrew and Wu et al. [39, 40]. Furthermore, downregulated DEP CD99, a critical regulator of leukocyte TEM, participates in the initiation of an inflammatory immune response [41]. Therefore, this study could conclude that the activation of the inflammatory response is closely related to the pathogenesis of ESRD.

### GO and KEGG functional enrichment analyses of DMPs

**Fig. 4:**
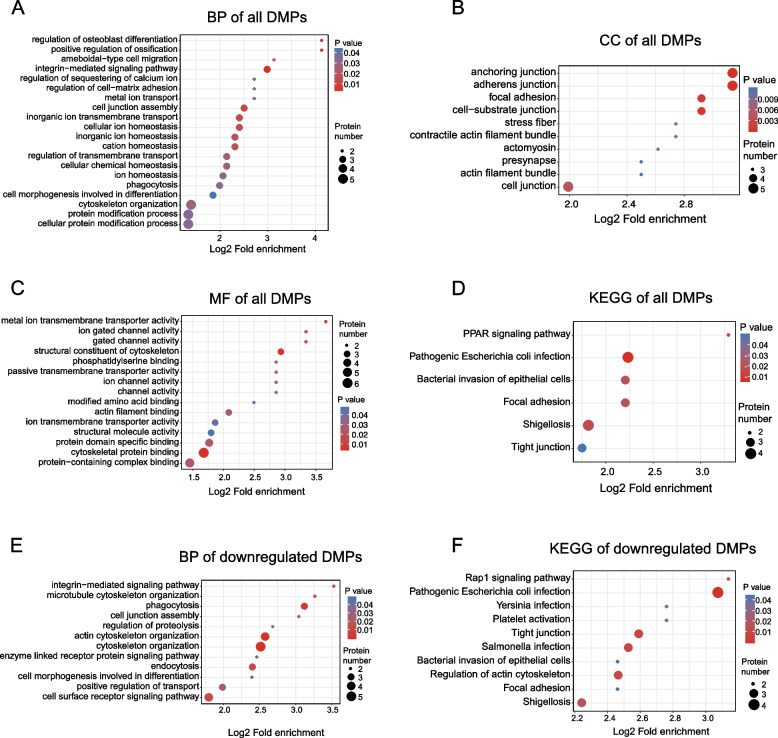
Gene Ontology (GO) and Kyoto Encyclopedia of Genes and Genomes (KEGG) functional enrichment analysis of all differentially malonylated proteins (DMPs) and down-regulated DMPs in end-stage renal disease (ESRD) patients compared with normal controls (NCs). **A** Biological process (BP) analysis of all DMPs. **B** Cellular component (CC) analysis of all DMPs. **C** Molecular function (MF) analysis of all DMPs. **D** KEGG functional enrichment analysis of all DMPs. **E** BP analysis of down-regulated DMPs. **F** KEGG functional enrichment analysis of down-regulated DMPs. The size of the circles denotes the number of DMPs enriched in the pathway, while circle color indicates the *P-*value significance (larger circle and red color indicates stronger significance)

This research performed functional enrichment analyses on DMPs and identified the main GO terms and six notable KEGG pathways (Fig. 4 and Supplementary Table 5). The collected datasets were subjected to a GO enrichment analysis in the context of BP, MF, and CC. For BP, DMPs were mainly enriched in a variety of processes, including positive regulation of ossification, cytoskeleton organization, actin cytoskeleton organization, cell surface receptor signaling pathway, regulation of osteoblast differentiation, and integrin-mediated signaling pathway (Fig. 4A). For CC analysis, adherens junction, anchoring junction, and focal adhesion were enriched (Fig. 4B). While for MF, the enriched DMPs were protein-containing complex binding, cytoskeletal protein binding, actin filament binding, and structural components of the cytoskeleton (Fig. 4C). Regarding the analysis of KEGG enrichment, this study identified higher levels in pathways involved in the PPAR signaling pathway, focal adhesion, pathogenic *Escherichia coli* infection, shigellosis, bacterial invasion of epithelial cells, and tight junction (Fig. 4D).

This study also evaluated the functions of the downregulated DMPs. The terms most prevalent in the BP categories among these annotations were cytoskeleton organization, actin cytoskeleton organization, and phagocytosis integrin-mediated signaling pathway (Fig. 4E). Meanwhile, the KEGG analysis suggested that the enriched terms included negative regulation of the Rap1 signaling pathway, platelet activation, tight junction, regulation of the actin cytoskeleton, focal adhesion, etc. (Fig. 4F). Moreover, other KEGG pathways included shigellosis, pathogenic E. coli infection, and bacterial invasion of epithelial cells, suggesting that pathogenic microorganism infection may also be linked to ESRD pathogenesis.

**Fig. 5:**
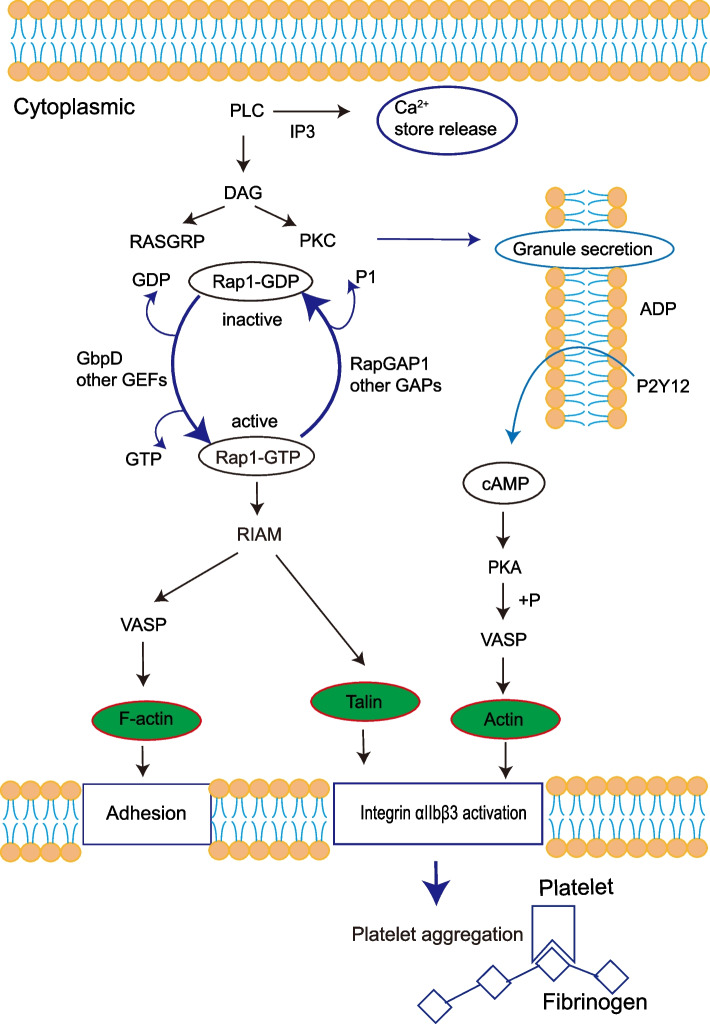
The complex signaling network represents the combination of Kyoto Encyclopedia of Genes and Genomes (KEGG) pathway Rap1 signaling pathway and Platelet activation (extracted from the KEGG database, hsa04051, hsa04611) related to malonylated proteins in the end-stage renal disease (ESRD) patients when compared to normal controls (NCs). The interaction map comprises the differentially malonylated proteins (green filled red bordered boxes) and were integrated in the regulation of focal adhesion and integrin αIIbβ3 activation. Rap1b-mediated inside-out activation of integrin αIIbβ3 involves the Rap1 effector RIAM and the cytoskeletal proteins talin and kindlin. In turn, integrin αIIbβ3 binding to fibrinogen stimulates an outside-in signaling able to promote Rap1b activation, which is an essential step for platelet spreading on fibrinogen. Malonylated proteins colored in green shades (down-regulation) are enriched in Rap1 signaling pathway and Platelet activation pathway. Abbreviations for the discussed proteins: RAP1: Ras-related protein Rap-1; RIAM: Rap1-interacting adaptor molecule; ADG: diacylglycerol; PLC: phospholipase C; VASP:vasodilator-stimulated phosphoprotein

In light of the findings of KEGG enrichment analysis, this study discovered that a large number of DMPs were abundant in the inflammatory-related signaling pathways, such as the Rap1 signaling pathway and the platelet activation pathway, both of which contained the downregulated DMPs, TLN1 and ACTB (Fig. 5). This is consistent with the conclusion that the Rap1 signaling pathway plays a critical role in platelet-integrin activation and platelet production.

### Motif analysis of the malonyl sites

**Fig. 6:**
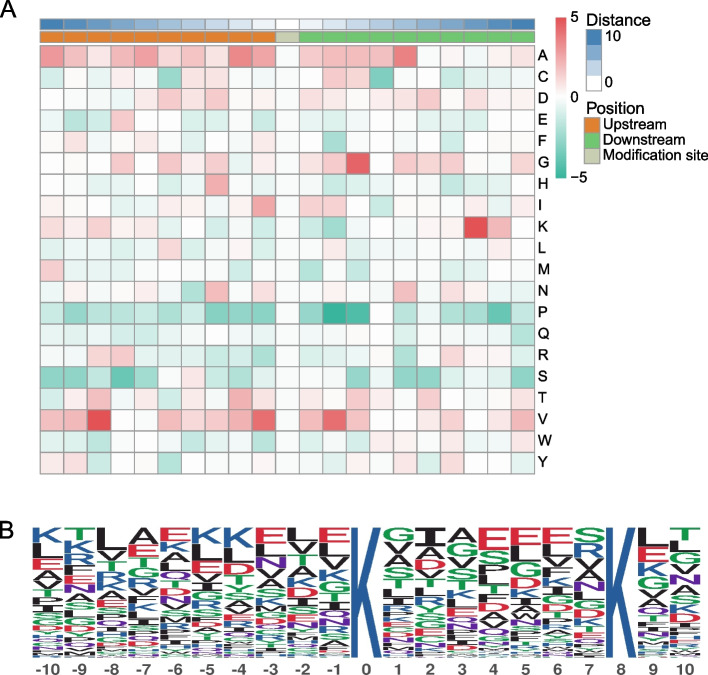
Motif Analysis of the Malonylation sites. **A** Motif enrichment heat map of all identified malonylation modification sites. Red indicates significant enrichment of the amino acid near the modification site, while green indicates significant reduction of the amino acid near the modification site. **B** Significantly enriched malonylation motifs extracted from the overrepresented malonylpeptide dataset by Motif-X. In total, one enriched malonylation site motif was identified

Using the Motif-X tool, this study searched for malonylation motif sites surrounding the identified malonylation sites (10 amino acids upstream and 10 amino acids downstream of each malonylation site) of the lysine residues (Fig. 6A). KxxxxxxxK (Fig. 6B) was a conserved motif discovered that encompassed the Kmal sites with a motif score of 6.61 and a fold increase of 1.7. The concentration or depletion of certain amino acids near the lysine malonylation sites was also depicted using a heat map. Amino acids such as alanine (A), glycine (G), lysine (K), and valine (V) were frequently found around serine malonylation sites. However, proline (P) and serine (S) amino acids were observed to be lower in the vicinity of the Kmal site. The favored amino acids around the malonylation sites represent the enzymes that catalyze the peculiar malonylation recognition in the PBMC of patients with ESRD. Overall, the above findings revealed that these motifs could disclose malonylation-related features in individuals with chronic renal failure.

## Discussion

Post-translational modifications are essential for increasing the functional diversity of proteins. They significantly influence protein function in health and disease [42]. Malonylation is a newly discovered and evolutionarily conserved alteration [26, 43] that remains poorly understood. Although malonylation has been found in a variety of metabolic pathways [28], studies on its role in the development of renal diseases are sparse. Galván-Peña et al. identified that activated macrophages enabled the synthesis of pro-inflammatory cytokines by GAPDH. The enzymatic activity of this GAPDH was increased by malonylation [29]. On the basis of these studies, the current study performed label-free quantification proteomics to investigate the possible involvement of lysine malonylation in the process of uremia.

CKD is related to inflammation. Thus, platelet activation and aggregation, in turn, influence kidney inflammation. By far, patients with lower eGFR were associated with a more vigorous inflammatory response and platelet cell dysfunction secondary to uremic toxins and pro-inflammatory markers [44, 45]. However, a study by Zhao et al. explains platelet hyperactivity in mice with CKD. They claim increased platelets due to the increase in serum indoxyl sulfate levels [46]. These factors work together to influence inflammation and platelet dysfunction, which progresses to the point of thrombotic and bleeding disorders in patients with ESRD [47].

Despite previous attempts, its pathogenic processes remain unknown and necessitate further research. Therefore, proteomics has emerged as a critical field of study for unlocking vital insights into the development of ESRD. In this paper, DEPs were shown to be involved in a variety of biological processes related to inflammation and immunity. Notably, most down-regulated DEPs were related to signal transduction, signal molecules, and interaction. They also participated in leukocyte TEM and complement and coagulation cascade pathways. Simultaneously, this study has observed 12 DMPs in patients with ESRD according to proteomic tools. DMPs, TLN1, and ACTB, were significantly decreased in PBMC, and both are involved in the Rap1 signaling pathway and platelet activation through KEGG pathway analyses (Fig. 5). The GO functional enrichment study of DMP revealed that DMPs play a major role in the integrin-mediated signaling pathway in patients with ESRD. In addition to this, KEGG functional analysis revealed that down-regulated DEPs and DMPs were abundant in the Rap1 signaling pathway, implying a strong link between these proteins and the ESRD signaling processes.

TLN1 (Talin-1) is a protein that binds integrins to the actin cytoskeleton. It also regulates cell migration and focal adhesion signaling. It is found in the adhesion complex between cells and the extracellular matrix (ECM) [48]. Intracellular levels of TLN1 are thought to trigger integrin activation [49]. Therefore, integrins mediate the extravasation of leukocytes to the sites of inflammation [50, 51]. RIAM (Rap1-interacting adaptor molecule) was discovered to be a Rap1-binding protein that is a critical component on the path to leukocyte-integrin activation [52]. RIAM binds to membrane-bound Rap1-GTP via its Ras association domain [53]. Additionally, TLN is connected to RIAM in its N-terminal region [54]. On the other hand, RIAM also interacts with PLCγ1, profilin, and VASP (vasodilator-stimulated phosphoprotein) through proline-rich areas [55, 56]. As a result, RIAM acts as a scaffold that binds the membrane targeting sequences of Rap1 to TLN. Therefore, TLN is attracted to the plasma membrane and activates the integrins [57]. The Rap1-RIAM-Talin complex appears to be a pivot point in αIIbβ3 integrin activation, involving actin remodeling, cytoskeletal reorganization, and signal integration for activation [58, 59]. After the recruitment of active Rap1, its downstream effectors RIAM and TLN are brought to the plasma membrane, and integrin signaling can be triggered [60]. The Rap1/RIAM/TLN1 pathway regulates the activity of various integrin classes expressed on platelets and neutrophils, both of which rely on integrin-mediated responses [61]. This study discovered that downregulated TLN1 in ESRD patients undergoes malonylation of lysine 2024 in its catalytic domain via a Rap1/RIAM/TLN1 pathway, which might affect integrin signaling. In peritoneal neutrophils, T.J.F. Lim and I.H. Su found that TLN1/K2454 trimethylation was induced in vivo during the course of peritonitis. Treatment with an Ezh2 inhibitor demonstrated the relevance of this methylation in controlling neutrophil infiltration into the peritoneal cavity [62]. In general, the findings of this investigation suggested that TLN1 malonylation appeared to alter downstream integrin signaling. As a result, reduction of TLN1 expression in platelets and leukocytes prevented αIIbβ3 activation, resulting in severe bleeding and leukocyte adhesion problems, both of which were associated with the progression of ESRD.

The ACTB gene is a cytoplasmic actin isoform that encodes an abundant cytoskeletal housekeeping protein, β-actin, and controls cell growth and migration [63, 64]. A previous study speculated that the ACTB gene could play a role in epithelial-to-mesenchymal transition (EMT)-induced diabetic kidney disease (DKD) [65, 66]. EMT-induced DKD is also the most common primary cause of ESRD. Additionally, a study presented by Liu et al. described ACTB methylation in the blood as a marker for the assessment and preclinical identification of stroke risk [67]. Torres-Gomez et al. proposed that RIAM, through its interaction with VASP, serves as a crucial organizer in the coordination of β2 integrin-induced actin cytoskeletal dynamics. The phosphorylation of VASP at Ser157 showed an increase in complement-mediated phagocytosis and a partial reversion of the RIAM knockdown phenotype, conveying conformational changes in integrin to F-actin [68]. Consistent with the above findings, this research similarly showed that decreased malonylation of ACTB at lysine61 in patients with ESRD could regulate its upstream effector RIAM by recruiting different binding partners. This supports the proposal that the blood-based ACTB hypo-malonylation may serve as a potential biomarker to help in the development of kidney disease.

## Conclusions

Inflammation is characterized by the migration of leukocytes into a tissue. Leukocyte integrins have emerged as promising therapeutic targets in various inflammatory diseases due to their crucial involvement in leukocyte recruitment. Furthermore, certain anti-leukocyte integrin therapies have also reached clinics [69]. This study proposes that by engaging the Rap1 signaling pathway and platelet activation, hypomalonylation of TLN1 and ACTB may have an association with the progression of ESRD. To conclude, the malonylated proteins TLN1 and ACTB were down-regulated and therefore inactivated integrin-mediated responses. This promoted platelet activation and inflammation, to some extent, which eventually influenced the progression of ESRD. These findings help broaden our understanding of the inflammatory pathway and platelet functions in patients with ESRD. The non-invasive proteomic analysis of PBMC performed in this study serves as a treatment target for kidney diseases; however, it has certain limitations to consider. These findings may not be generalizable due to the small and limited sample size. Although the findings appear credible, further research and additional experiments are required to verify them.

### Supplementary Information

**Additional file 1. **

**Additional file 2. **

**Additional file 3. **

**Additional file 4. **

**Additional file 5. **

**Additional file 6. **

**Additional file 7. **

**Additional file 8: Supplementary Table 1.** The LFQ intensities for the quantitated proteins. **Supplementary Table 2:** Information of 793 DEPs (FC<1/1.5 or FC>1.5 and *P*<0.05). **Supplementary Table 3:** Information of 12 DMPs (FC<1/1.5 or FC>1.5 and *P*<0.05). **Supplementary Table 4:** GO functional fold enrichment of DEPs. **Supplementary Table 5:** GO functional fold enrichment of DMPs.

## Data Availability

The mass spectrometry proteomics data have been deposited to the ProteomeXchange Consortium via the PRIDE partner repository with the dataset identifier PXD035147.

## Abbreviations

ESRD: End-stage renal disease
PBMCs: Peripheral blood mononuclear cells
LC–MS/MS: Liquid chromatography-tandem mass spectrometry
PTMs: Posttranslational modifications
DEPs: Differentially expressed proteins
CKD: Chronic kidney disease
eGFR: Estimated glomerular filtration rate
RRT: Renal replacement therapy
MHP: Patients on maintenance hemodialysis
WT: Wild-type
ALDOB: Aldolase B
DMPs: Differentially malonylated proteins
Kmal: Lysine malonylation
FC: Fold change
GO: Gene ontology
CC: cellular component
BP: Biological process
MF: Molecular function
KEGG: Kyoto Encyclopedia of Genes and Genomes
KDIGO: Kidney Disease Improving Global Outcomes Organization
PBS: Phosphate buffered saline
TCA: Trichloroacetic acid
DTT: Dithiothreitol
IAA: Iodoacetamide
UHPLC: Ultra-high-performance liquid chromatography
LCMS: Liquid chromatography mass spectrometry
FDR: False discovery rate
GWAS: Genome-wide association studies
TEM: Leukocyte transendothelial migration
ECM: Extracellular matrix
RIAM: Rap1-interacting adaptor molecule
VASP: Vasodilator-stimulated phosphoprotein
EMT: Epithelial-to-mesenchymal transition
DKD: Diabetic kidney disease
