# Supplementary figures and images for: Integrated proteome and malonylome analyses reveal the potential meaning of TLN1 and ACTB in end-stage renal disease
Source: Proteome Sci. 2023 Oct 13;21:18. doi: 10.1186/s12953-023-00211-y (PMC10571336; doi:10.1186/s12953-023-00211-y)

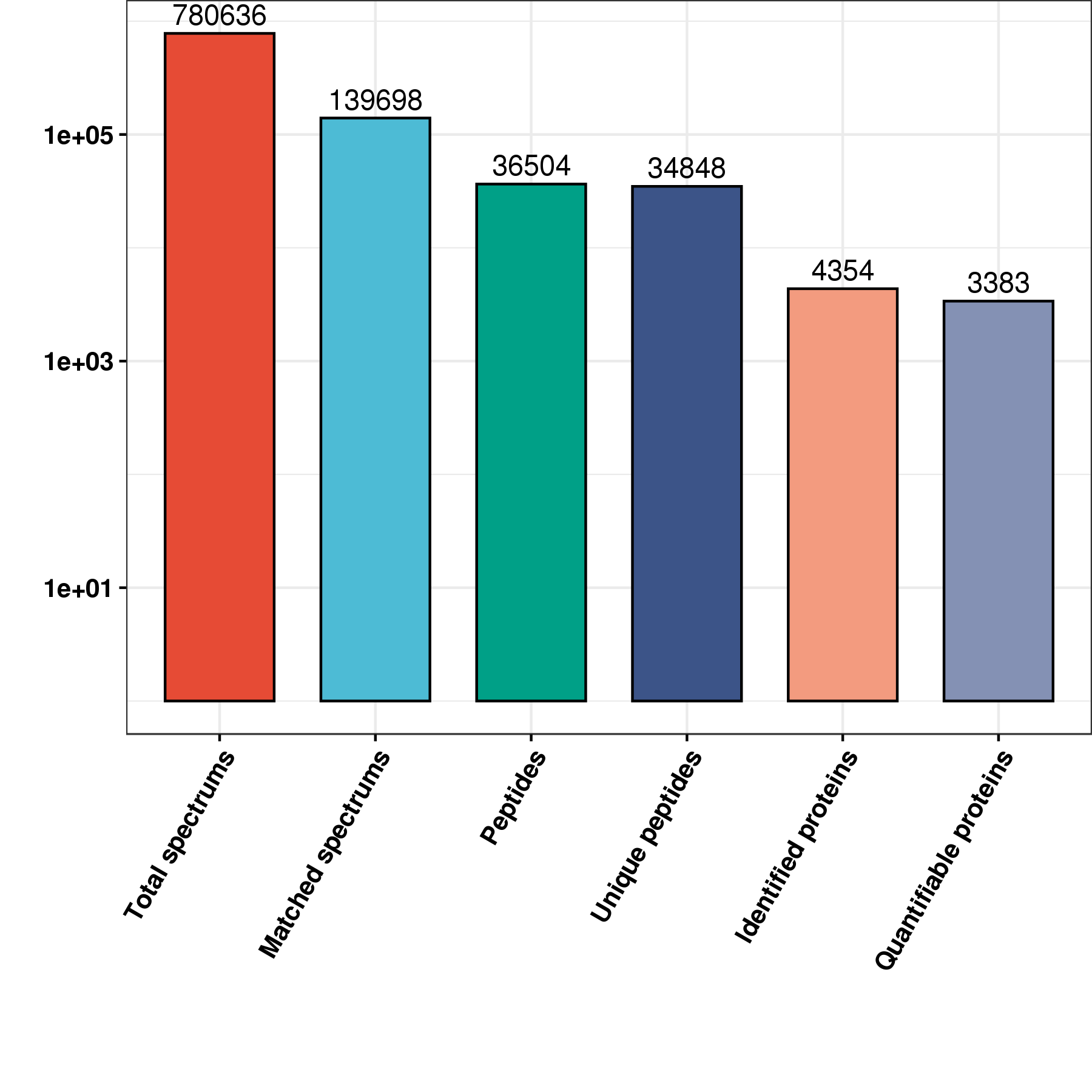

Supplement: Supplementary file 1 — Additional file 1. [file 12953_2023_211_MOESM1_ESM.png]

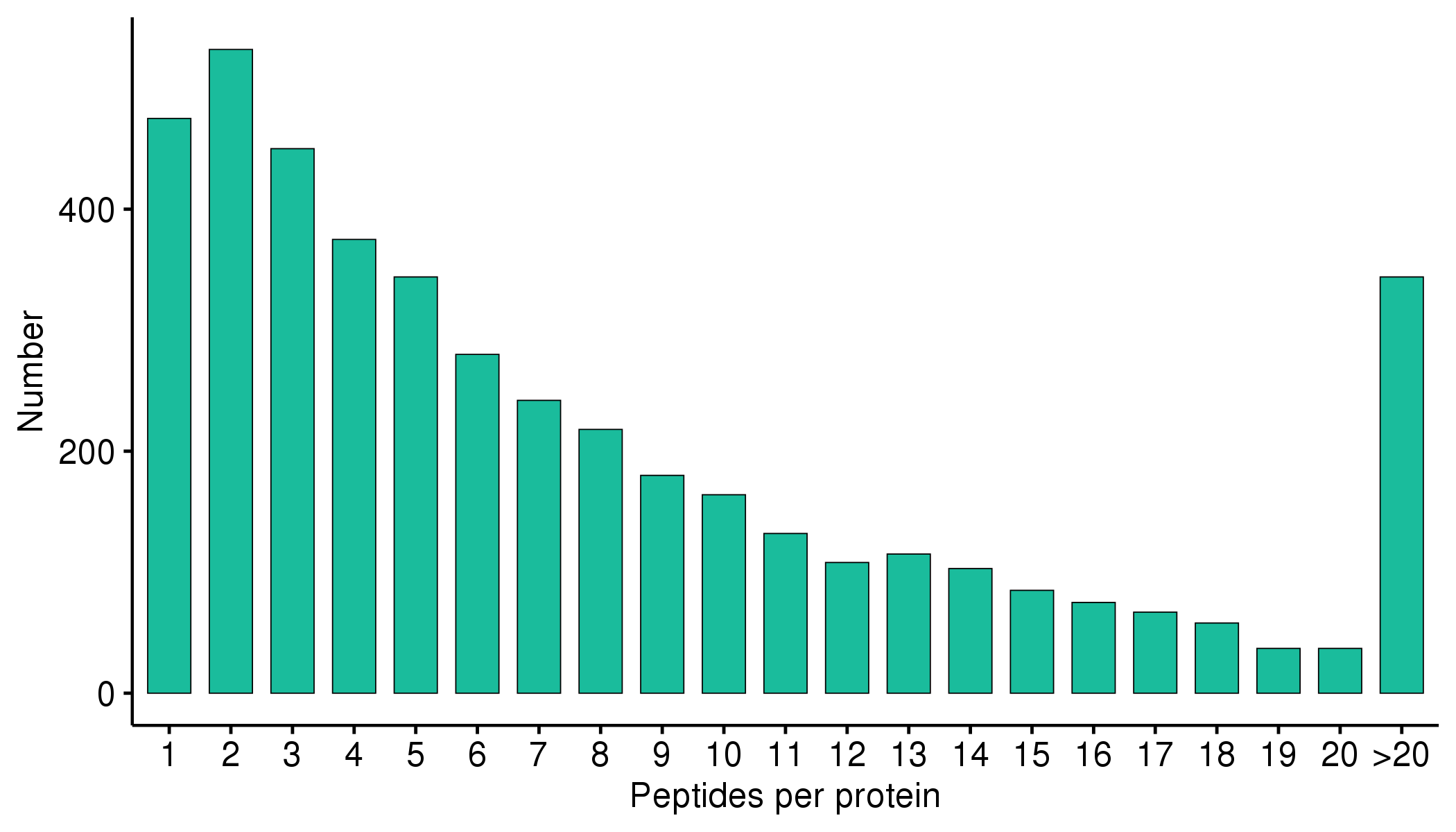

Supplement: Supplementary file 2 — Additional file 2. [file 12953_2023_211_MOESM2_ESM.png]

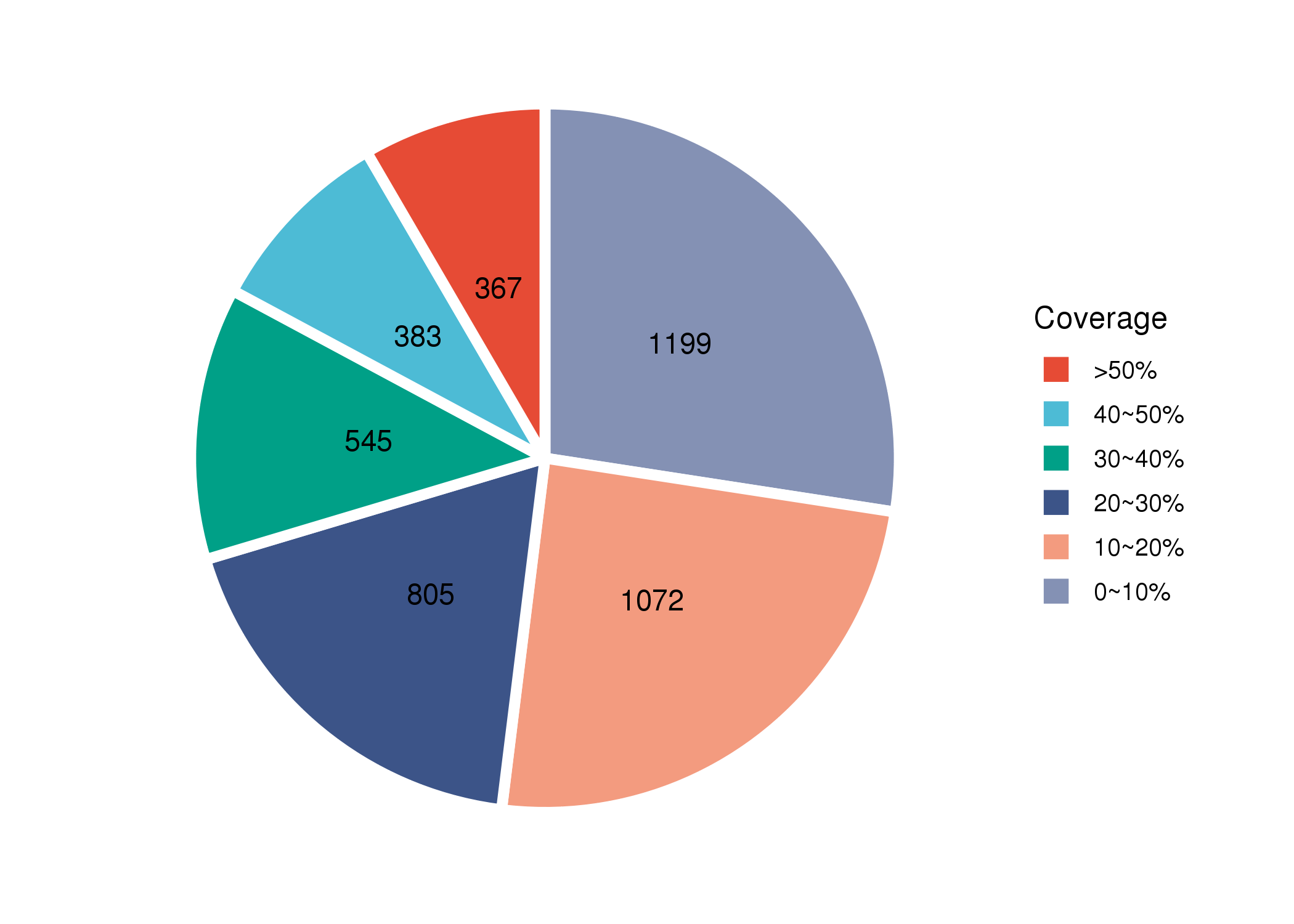

Supplement: Supplementary file 3 — Additional file 3. [file 12953_2023_211_MOESM3_ESM.png]

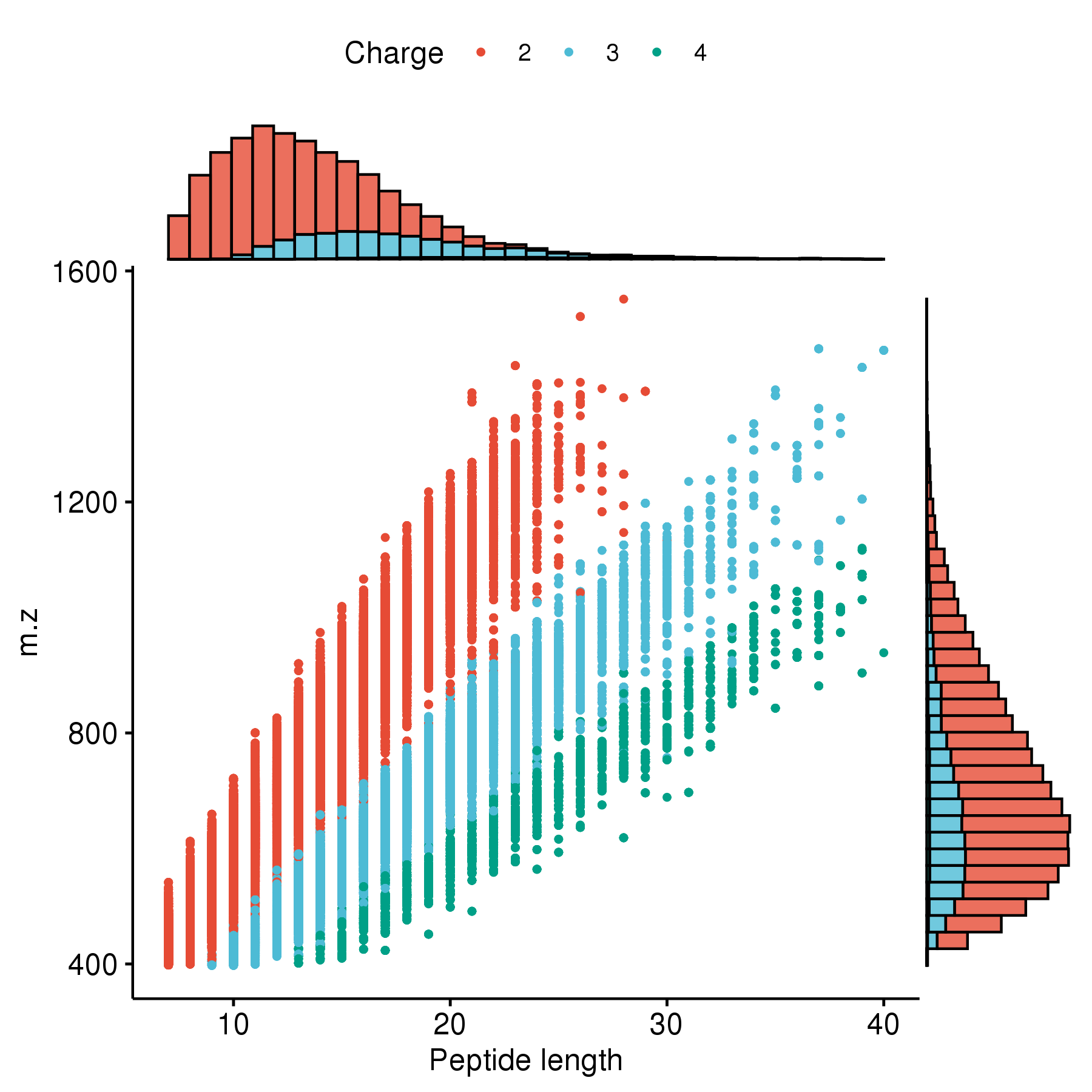

Supplement: Supplementary file 4 — Additional file 4. [file 12953_2023_211_MOESM4_ESM.png]

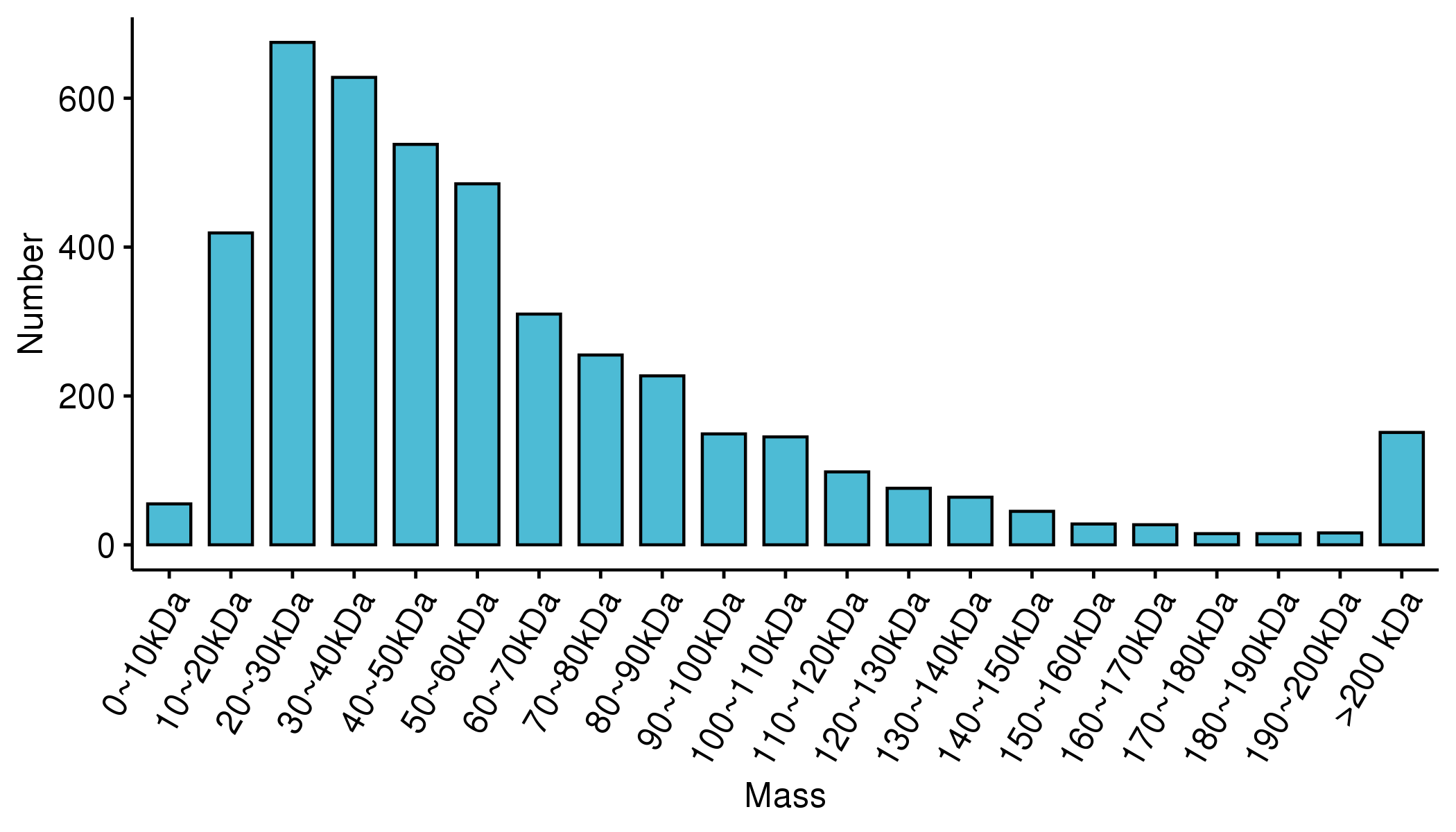

Supplement: Supplementary file 5 — Additional file 5. [file 12953_2023_211_MOESM5_ESM.png]

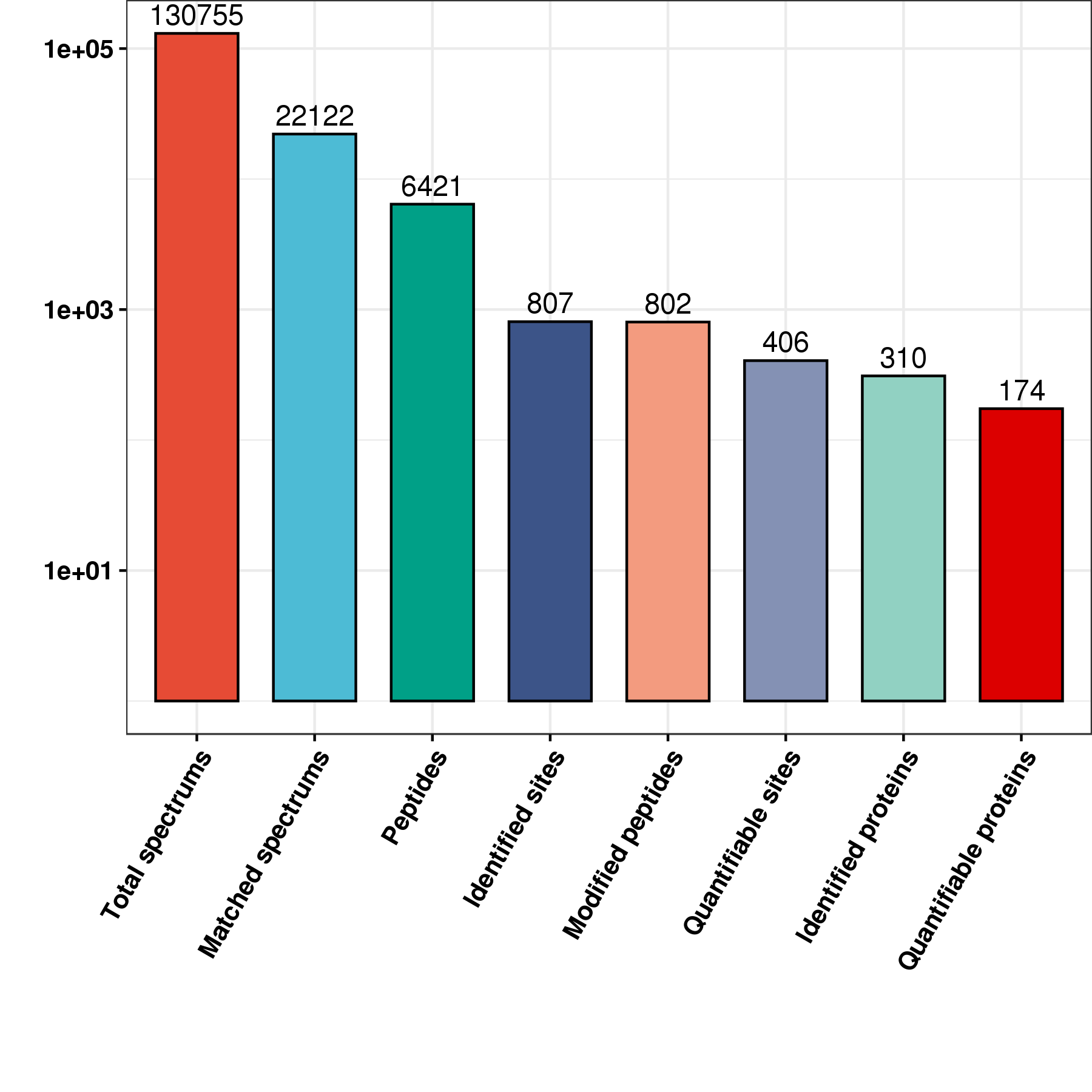

Supplement: Supplementary file 6 — Additional file 6. [file 12953_2023_211_MOESM6_ESM.png]

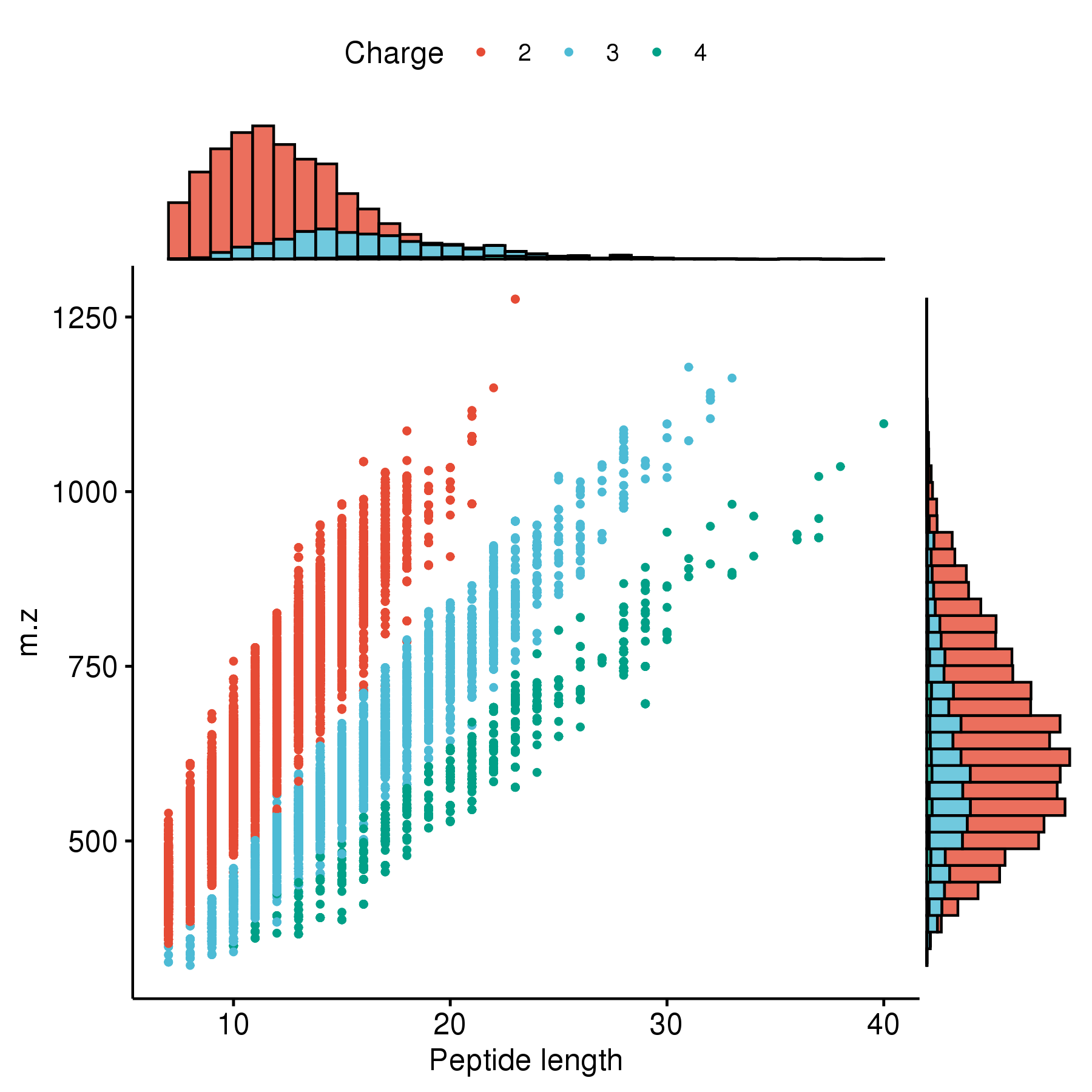

Supplement: Supplementary file 7 — Additional file 7. [file 12953_2023_211_MOESM7_ESM.png]
